# Continuous Intravenous Administration of Granulocyte-Colony-Stimulating Factors—A Breakthrough in the Treatment of Cancer Patients with Febrile Neutropenia

**DOI:** 10.3390/medicina57070675

**Published:** 2021-06-30

**Authors:** Călin Căinap, Sânziana Cetean-Gheorghe, Laura Ancuta Pop, Daniel Corneliu Leucuta, Doina Piciu, Andra Mester, Cătălin Vlad, Crişan Ovidiu, Alexandra Gherman, Cristina Crişan, Alina Bereanu, Ovidiu Bălăcescu, Anne Marie Constantin, Irina Dicu, Loredana Bălăcescu, Adina Stan, Patriciu Achimaş-Cadariu, Simona Căinap

**Affiliations:** 1 Faculty of Medicine, “Iuliu Hațieganu” University of Medicine and Pharmacy, 400000 Cluj-Napoca, Romania; calincainap2015@gmail.com (C.C.); dleucuta@umfcluj.ro (D.C.L.); doina.piciu@umfcluj.ro (D.P.); piciuandra@gmail.com (A.M.); catalinvlad@yahoo.it (C.V.); allexandragherman@gmail.com (A.G.); amc@gmail.com (A.M.C.); adina@ssnn.ro (A.S.); pachimas@umfcluj.ro (P.A.-C.); sorana.cainap@umfcluj.ro (S.C.); 2 Ion Chiricuta Institute of Oncology, 400015 Cluj-Napoca, Romania; cristina_persa@yahoo.fr (C.C.); ovidiubalacescu@iocn.ro (O.B.); ruirina@yahoo.com (I.D.); loredana_balacescu@yahoo.com (L.B.); 3 Faculty of Pharmacy, “Iuliu Hațieganu” University of Medicine and Pharmacy, 400000 Cluj-Napoca, Romania; ocrisan@umfcluj.ro; 4 Research Center for Functional Genomics, Biomedicine and Translational Medicine, University of Medicine and Pharmacy Iuliu Hatieganu, 400000 Cluj-Napoca, Romania; laura.pop@umfcluj.ro; 5 Faculty of Medicine, “Lucian Blaga” University of Sibiu, 550024 Sibiu, Romania; alinabereanu@gmail.com

**Keywords:** neutropenia, chemotherapy, cancer, G-CSF, febrile

## Abstract

*Background*: Febrile neutropenia (FN) remains one of the most challenging problems in medical oncology and is a very severe side effect of chemotherapy. Its late consequences, when it is recurrent or of a severe grade, are dose reduction and therapy delays. Current guidelines allow the administration of granulocyte-colony-stimulating factors (G-CSF) for profound FN (except for the case when a pegylated form of G-CSF is administrated with prophylactic intention) in addition to antibiotics and supportive care. *Methods*: This is a prospective study that included 96 patients with confirmed malignancy, treated with chemotherapy, who developed FN during their oncological therapy, and were hospitalized. They received standard treatment plus a dose of G-CSF of 16 µg/Kg/day IV continuous infusion. *Results*: The gender distribution was almost symmetrical: Male patients made up 48.96% and 51.04% were female patients, with no significance on recovery from FN (*p* = 1.00). The patients who received prophylactic G-CSF made up 20.21%, but this was not a predictive or prognostic factor for the recovery time from aplasia (*p* = 0.34). The median chemotherapy line where patients with FN were included was two and the number of previous chemotherapy cycles before FN was three. The median serological number of neutrophils (PMN) was 450/mm^3^ and leucocytes (WBC) 1875/mm^3^ at the time of FN. Ten patients possess PMN less than 100/mm^3^. The median time to recovery was 25.5 h for 96 included patients, with one failure in which the patient possessed grade 5 FN. Predictive factors for shorter recovery time were lower levels of C reactive protein (*p* < 0.001) and procalcitonin (*p* = 0.002) upon hospital admission and higher WBC (*p* = 0.006) and PMN (*p* < 0.001) at the time of the provoking cycle of chemotherapy for FN. The best chance for a shorter duration of FN was a short history of chemotherapy regarding the number of cycles) (*p* < 0.0001). *Conclusions*: Continuous IV administration of G-CSF could be an alternative salvage treatment for patients with profound febrile neutropenia, with a very fast recovery time for neutrophiles.

## 1. Introduction

Cancer is a disease described from ancient times and many attempts to treat it have been conducted. From initial natural ointments, pastes, herbal solutions to actual synthetic treatments such as cytotoxic and hormonal agents, targeted therapy, and immunomodulatory therapies, many attempts were made to cure this disease. Whether the attempts were natural or synthetic, all of them have side effects.

The last decades showed an increased survival rate of cancer patients due to extensive research performed in oncology; With the emergence of new and effective drugs, the added survival benefit approximated to be at least 21% compared to those with surgery performed alone [1]. This prolonged life expectancy for oncologic patients has come with a price—increased toxicities, most of them linked to the fraction of multiplication of the cells of specific tissues or organs. Bone marrow and intestinal mucosa are most frequently at risk as they are the unwanted targets of classical chemotherapy. Fifty percent of patients with solid tumors and 80% of those with hematological malignancies are at risk for febrile neutropenia when treated with systemic chemotherapy [2]. Unfortunately, the prognostic of grade 4 neutropenia toxicity is very drastic, with mortality that can reach as high as 50% if the septic shock is present [2]. A long-term consequence of hematologic toxicities could be represented by delays of the treatment, reduced doses for systemic oncological regimens with consecutive lack of response and efficacy, or even therapy discontinuation.

The burden of post-chemotherapy febrile neutropenia is represented by infections that could endanger the patients’ lives, increase treatment costs, decrease in the quality of life, and lower life expectancy.

Managing febrile neutropenia can be a very difficult task depending on what the oncologist has at his disposal in terms of logistics—imaging department, bacteriological laboratory, pharmacy, intensive care unit, and access to a specialist in infectious diseases.

The discovery of granulocyte colony-stimulating factor (G-CSF) represents a big breakthrough in changing the prognostic, natural history, and lifesaving of oncological patients with severe postchemotherapy toxicity, which could become a therapeutical emergency such as deep febrile neutropenia. The role of G-CSF in preventing aplasia for patients with systemic chemotherapy is well known and it is indicated in current guidelines [3]. Concerning the use of G-CSF during the aplastic period, no consensus has been reached until recently. Despite the lack of proofs regarding the survival advantage for aplastic patients in a critical clinical condition, such as grade 4 neutropenia, most oncologists use to administrate G-CSF to shorten the period of patient’s maximum risk for infection, hospitalization, and use of antibiotics. Beginning in 2015, NCCN updated guidelines permitting the administration of myeloid growth factors only if aplasia occurs after prophylaxis with G-CSF (with the exception of the pegylated form of G-CSF), which is, however, not included in the ESMO guidelines [3,4].

## 2. Materials and Methods

The study design was submitted for approval to the institutional Ethics Committee; the approval must be obtained as part of the institutional procedure for the Oncology Institute of Cluj-Napoca, Romania (number: 42/8 December 2015). The study was performed and respected the principles and recommendations of the Declaration of Helsinki. Before any oncological treatment or blood sample prelevations, all patients signed and provided informed consent. All data (personal or medical) were anonymized before and during all steps of the study in accordance with the General Data Protection Regulations.

The inclusion criteria:

- Age ≥ 18 years;
- Patients presenting histologically with confirmed solid tumor or hemopathy;
- Patients being treated with chemotherapy (regardless of the cycle);
- Patients who were prescribed treatment with G-CSF as adjunctive therapy for neutropenia (prophylaxis with G-CSSF was allowed);
- Fever is defined per institutional protocol as an oral or axillary temperature above 38 °C, with a presumed infectious etiology (even non-documented by positive bacteriological cultures) in the absence of paraneoplastic or non-infectious causes, for example, blood transfusion;
- Neutropenia (granulocyte count < 500/mm^3^) induced by curative or palliative chemotherapy regimens, without a cause of bone marrow failure;
- Treatment as an inpatient, with antibiotic regimen (per institutional protocols).
- The exclusion criteria:
- Patients with prior chronic or acute antibiotic therapy for a bacterial infection;
- Patients with shock (whatever the etiology) (systolic blood pressure less than 90 mm Hg, less responsive to treatment peripheral perfusion, and coma or altered mental status);
- Patients subject to a bone marrow transplantation procedure;
- Patients with severe renal failure or impairment (creatinine clearance rate < 15 mL/min/1.73 m^2^ surface body);
- Patients with abnormal liver function (transaminases elevated more than five times compared with the upper limit of normal or bilirubin more than 3 mg/dL);
- Patients with pregnancy positive test or breast-feeding;
- Patients allergic to antibiotics or any of the ingredients of G-CSF product;
- Patients presenting with a myelodysplastic syndrome;
- Patients not treated with chemotherapy;
- Patients that were included and excluded from a clinical trial less than 90 days from the actual study.

**Study objectives**

1. Shortening the recovery time from febrile neutropenia.

This is a prospective study; 96 patients were enrolled between 2015 and 2018. Following the institutional protocol, all patients were admitted to the hospital. Each patient signed informed consent before any procedures or inclusion in the study. During the aplasia period, daily blood samples were taken in accordance to the institutional protocol. From their medical records, we assessed some clinical and laboratory results which could influence the length of chemotherapy-induced neutropenia. These include demographic data, baseline lab tests (one cycle before aplasia, starting date of neutropenia, and daily lab samples taken according to institutional protocol), treatment intention, and data regarding treated neoplasia (type of the tumor and stage).

The length of the aplasia was established by considering the date of the diagnostic and the date of the resolution. The length in minutes was calculated by taking into consideration the hour and minute of the blood sample registration to the internal lab informatics IT system.

Descriptive statistics were assessed using counts and percentages for categorical and continuous data and means (for normally distributed data), medians, interquartile ranges, and ranges for quantitative data that did not follow the normal distribution. The bias-corrected and accelerated bootstrapped 95% confidence intervals were computed for the main variables of interest. Comparisons between two independent groups regarding non-normal quantitative data were performed with Wilcoxon rank-sum test. R.I. statistical analysis was performed using Excel 2010 and R version 3.5.1 Microsoft Windows version 7. A two-tailed *p*-value of <0.05 was statistically significant. 

## 3. Results

**Figure 1:**
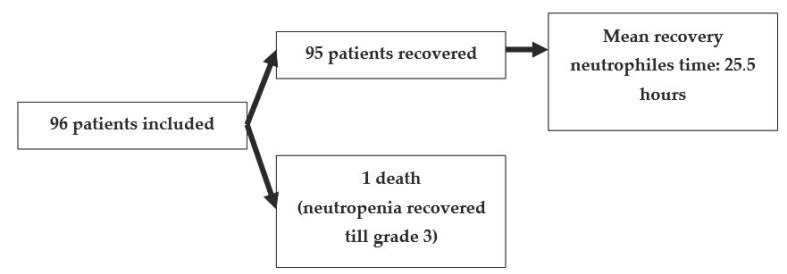
Chart of the resolution of FN for the included population.

From the initial population of 96 included patients, 95 recovered from grade 4 aplasia, with only one death event in a patient with initially severe neutropenia (<100/mm^3^) which was ameliorated to grade 3 following the study protocol as detailed in Figure 1.

In Table 1, we describe the main clinical characteristics of the patients who were considered eligible for the study, with a focus on items that could determine prolonged post-chemotherapy aplasia according to the main published data.

**Figure 2:**
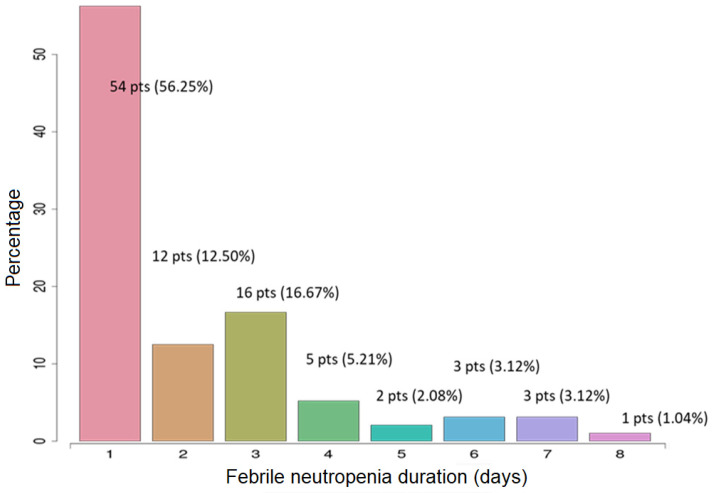
Repartition of aplasia among the included patients.

Considering the large scale of clinical use of G-CSF in neutropenic patients, the innovative aspect of the study was to improve and assess the length of the aplastic episode with this new perfusion strategy of G-CSF. This period of aplasia varied between 1 and 8 days. Most FN episodes only lasted one day (56.25%) and 85.64% were resolved within less than 72 h. The exact numbers of patients with 1 day aplasia, 2 days, and so on are detailed in Figure 2.

The median duration of FN episodes in the included population, expressed in days and hours, is stated in Table 2. The differences in terms of indication of chemotherapy—curative disease or palliative treatment—are not statistically significant. The secondary prophylaxis of FN by using G-CSF did not seem to influence the length of aplasia under IV continuous administration of G-CSF.

## 4. Discussion

G-CSF is a recombinant stimulating factor for the growth and maturation of myeloid progenitor cells. For this action, it requires the presence of G-CSF receptors at different levels upon the differentiation in the neutrophilic lineage [5]. In vivo, the effect of G-CSF administration could be represented by a temporary fall (1–2 h) in peripheric neutrophiles number (increased margination effect) and followed by an increased number secondary to the release from the place of ‘production’—the bone marrow of mature granulocytes [5,6]. At the bone marrow level, G-CSF will stimulate the proliferation of neutrophils precursors, differentiation of pluripotent stem cells from bone marrow on the myeloid line, and release through sinusoids into the bloodstream [6,7]. In the bone marrow, the myeloid line consists of two-compartments: proliferative (precursors of granulocytes) and non-proliferative pool (mature cells ‘stock’) [8]. The total duration for neutrophiles development from initial pluripotent stem cell is approximately 14 days, with several stages of maturation from myeloblasts, promyelocyte, metamyelocytes, and mature neutrophil [7]. Promyelocyte is a dedicated precursor for neutrophils.

Granulocytes are under the control of several cytokines, such as CXCL12 (stromal cell-derived factor) and CXCL2 (macrophage inflammatory protein), both of them having the role of increasing the chemotactic signal and consequently stimulating the neutrophils to move into the bloodstream [9]. These cytokines are secreted by cells that are located in bone sinusoids (CXCL2) or endothelial cells (CXCL2) [9]. Opposing these signals is the CXCR4 (SDF1-a receptor) and it is described as a dominant retention signal [9]. All these cytokines have a common intracellular mediator of the Rho family, which are Rac1 and Rac2 [9]. By hyperactivation or depletion of Rac1 and 2, the neutrophils will migrate or not migrate from the bone marrow and they also seem to be involved in vesicular trafficking control [10].

G-CSF accelerates the mitosis and maturation process, increases the motility and elution from the bone marrow of the neutrophils, and another possible mechanism (other than those mentioned above) could be performed through CXCR2 [11]. Both forms—short and long-acting available forms of G-CSFs—have clearance at the neutrophiles level and for the short-acting form of G-CSF there is also a renal elimination, which imposes daily drug administration; otherwise, its effect disappears within 24 h [12]. For the pegylated form of G-CSF, the renal component of clearance is eliminated; therefore, it remains under the neutrophil mechanism of degradation, which explains why only one injection is enough for each chemotherapy cycle [3,13]. Moreover, due to its chemical structure, the short-acting form is more easily absorbed from the subcutaneous tissue and has higher specific (neutrophile-linked) and unspecific elimination, which explains its lower biodisponibility compared to the long-acting G-CSF [12].

Neutrophil recovery represents the main issue for FN in oncological patients. Neutropenia could be responsible for chemotherapy regimen administration delay, unintended dose reduction, and secondary response rate failure. Since the overall incidence of cancer is continuously rising, the need and exposure to systemic treatment will continue to represent an important issue for oncological patients [14].

**To administrate or not the G-CSF in FN?**

Febrile neutropenia is considered an oncologic emergency that endangers patients’ lives. In order to diminish the risk of FN, the main guidelines recommend the use of G-CSF as secondary prophylaxis, especially for high and intermediate-risk chemotherapy regimens [3,4]. Morbidity associated with FN is significant—20 to 30%—and the overall mortality for oncological patients with FN could be as high as 10% or more, which can be towards 50% if a severe condition such as septic shock occurs [2,3].

With respect to primary or secondary prophylactic administration, the vast majority of the guidelines strongly support and recommend the use of G-CSF [3,13,15,16].

In the management of FN, the data regarding the utility of G-CSF administration are scarce and usually related to a low number of patients. The objectives of these trials are not the same in terms of the investigated items, but the focuses are mainly the recovery time for neutrophils, length of hospitalization, and use of antibiotics. Shortening the recovery time by the administration of G-CSF was demonstrated by Yoshida et al. on 214 febrile neutropenic episodes; Ozkaynak on 67 pediatric oncological patients; Soda et al. on 33 patients; Carbonero et al. on 210 patients with FN; and Cochrane meta-analysis on 1335 patients [17,18,19,20,21]. In addition, G-CSF administration showed a short recovery from fever on 966 evaluable patients and withdrawal from antibiotics on 457 participants from the included trials [21].

Even today, using G-CSF as adjunctive therapy is not recommended by all oncological professional associations. ASCO does not recommend it for all patients and it is kept as an option mainly for those with profound aplasia (neutrophils below 100/mm^3^) or if the physician expects a prolonged period of aplasia (more than ten days) [13].

**What type of G-CSF to administrate in oncological patients with FN?**

For clinical use, the oncologists may prescribe either short or long-acting granulocyte colony-stimulating factors. Both types of myeloid stimulating agents are approved by Food and Drug Administration (FDA) and European Medicines Agency (EMA) for administration during an FN episode to reduce the duration of neutropenia [3,12,13,22,23]. The main difference between the two pharmacological forms is represented by a molecule of 20 kDa—polyethylene glycol—which facilitates the long life of peg G-CSF by eliminating the renal clearance [8]. Other mathematical models suggested that the long life of peg G-CSF is due to its minimal absorption rate [24]. The short-acting G-CSF was estimated to have the potential of a superior stimulation of the bone marrow [12].

**Dosage and duration of IV continuous administration of G-CSF in F.N.**

In our study, we administrated a continuous IV perfusion of G-CSF at a dosage level of 16 microgram/kg body/day. The main objective of the study—diminishing the recovery time from aplasia—was achieved. The median time for recovery for the included patients was 25.5 h, which is much lower than indicated in other published works.

The standard dose for G-CSF is 5 micrograms/kg body/day or 250 micrograms/sqm/day for Granulocyte/Macrophage Colony Stimulating Factor (GM-CSF) or 6 mg/day for pegylated G-CSF [5]. G-CSF should be administrated until neutrophiles recover to values above 1000/mm^3^ [3,13].

The median duration of post-chemotherapy aplasia is between 6–8 days [3]. Reduction in the period of FN is one of the most important factors to diminish the risk of toxic fatality for oncological patients. The relative hesitations in recommending the use of G-CSF during the period of FN episodes were due to published data that did not show an overall survival improvement for G-CSF administration. In clinical practice and despite this lack of scientific proof, most oncologists extensively prescribe G-CSFs. With the accumulation of new data, the meta-analysis of Cornes et al. managed to show a significant reduction in duration for profound neutropenia, for hospitalization, antibiotic consumption, and increased chances for hematologic recovery [25].

Mathematical models showed that subcutaneous administration is not capable of maintaining optimal serological levels of G-CSF due to the rapid clearance of the drug; this suboptimal stimulation could be surpassed by a twice-daily injection or by peg G-CSF formulations [24]. A higher level of G-CSF, which could be assured by the peg G-CSF or continuous intravenous administration, will establish a new and higher point of equilibrium in approximately 16 h [24]. Some published data are suggesting that the non-glycosylated form of G-CSF succeeded to rise at a higher point in terms of the serum level of G-CSF than the pegylated drug, but the effect is only transitional [26]. When the level of granulocytes is too low, such as below 50/mm^3^, neither peg G-CSF nor subcutaneous G-CSF could quickly reverse the neutropenia. For the pegylated form of G-CSF, asymmetry between the serum number of neutrophils and G-CSF levels is approximated to be reached within 15 days and deep aplasia requires a higher stimulation which could be assured by IV continuous perfusion [24]. For those patients who are not recovering the neutrophiles within 24 h, the authors increased the subcutaneous dose of G-CSF or IV continuous perfusion [24].

Regarding the dose of G-CSF, for healthy donors for bone marrow transplant (BMT) (allogenic), FDA and EMA recommend a standard dose of G-CSF of 10 micrograms/kg body/day, with a safe increase in the dose up to 12 or even 24 micrograms/kg body/day in continuous subcutaneous perfusion if needed [23]. Moreover, a dose of 16 micrograms/kg body is more efficient than ten micrograms/kg body for the mobilization of CD34 cells for allogenic BMT in healthy donors [27]. For patients with congenital neutropenia, the optimal dose for a good granulocyte response is variable between 0, 7, and 70 micrograms/kg body [27].

In a published phase 1 study for patients who were treated with high-dose chemotherapy followed by an autologous BMT, a 14 day continuous intravenous infusion of G-CSF was compared with the 4 h infusion. The levels of the G-CSF dose were between 4 and 32 microgram/kg/day [16]. G-CSF at a dosage between 15 and 20 microgram/kg/day could increase the stimulation of the hematopoietic bone marrow; a dose higher than 16 microgram/kg did not show any potential therapeutical advantage and a dose exceeding 64 microgram/kg body could be deleterious for neutrophiles recovery [28].

These differences could be explained by the molecular mechanism of neutrophil’s response to G-CSF stimulation, which is essentially antagonistically driven by CXCR4 or CXCR2-linked Rac regulators [29].

**Methods of administration for G-CSF?**

Both the short and long-acting forms of granulocyte-colony stimulating factors could be administrated intravenously or subcutaneously [6,8].

The pharmacokinetics of G-CSF is very complex since it is non-linear. It could be explained by the dependence of the absorption of G-CSF on the receptor-mediated endocytosis processes, renal clearance, and neutrophile serum levels [5]. Research was conducted in healthy adults who were injected subcutaneously with a progressive dose of G-CSF of 2, 5, 5, and 10 microgram/kg compared to the pharmacokinetics of the drug with an intravenous (30-min infusion) administration profile of 5 microgram/kg. G-CSF was found to exhibit similar absorption rates, with a biodisponibility of 69.1% [5].

On the other hand, in deep aplasia, the stimulation is not equal for both types of G-CSF and standard dose depending on the level of neutropenia. Mathematical modeling suggests that the magnitude of the effect of G-CSF could differ with respect to the grade of neutropenia 300–500/mm^3^, 50–300/mm^3^, and less than 50/mm^3^ upon the dose of G-CSF, which are considered standard for short-acting at five microgram/kg or long-acting at 100 microgram/kg in the subcutaneous or continuous intravenous administration of 10 microgram/kg/day [24].

A study published in 2014 included 120 patients with hematological malignancies who were randomized between standard subcutaneous dose administration and the same dose administered by IV bolus injection [30]. The mean time for neutrophiles recovery was significantly longer for G-CSF IV administration (7.9 days versus 5.4 days, respectively) [30]. This result could be interpreted as predictable since the used IV the dose is insufficient to sustainably stimulate the bone marro according to all published mathematical pharmacokinetics and dynamics approximation of G-CSF.

## 5. Conclusions

In conclusion, our prospective study showed that continuous IV administration of G-CSF with a high dose (16 micrograms/kg/day) could represent an effective alternative for neutropenic patients, especially for those without any response within 24 h to standard treatment or profound neutropenia.

## Figures and Tables

**Table 1:** Main characteristics of the enrolled patients.

Item	Number of Patients	*N* (%)
Age, mean	96	58.79
Gender	Male	47 (48.96)
	Female	49 (51.04)
BMI, median (IQR)	73	24.05 (21.68–28.1)
G-CSF prophylactic before FN episode	96	19/94 (20.21)
Type of cancer	colon/rectum	21 (21.87)
	gastric	13 (13.54)
	ovarian	10 (10.42)
	lung	10 (10.42)
	head and neck	11 (11.46)
	germinal tumors	5 (5.20)
	other	26 (27.09)
TNM initial stage of neoplasia	1	5 (7.14)
	2	15 (21.42)
	3	21 (30)
	4	29 (41.42)
Chemotherapy with FN episode	line	2
	cycle	3.6
Chemotherapy regimen	96	
	platinum-based	48 (50)
	taxane-based	17 (17.70)
	antracycline	25 (26.04)
	other	6(6.25)
Disease status	controlled	2 (2.08)
	evolutive	94 (97.92)

BMI = body mass index; G-CSF = granulocyte-colony-stimulating factors; FN = febrile neutropenia; TNM = tumor, nodes, and metastases classification; IQR = interquartile range.

**Table 2:** Median duration of FN episodes.

Item	Median (IQR)	95% CI	Range
aplasia duration in days	1 (1–3)	1–2	1–8
aplasia duration	1530 (1449.75–4310.75)	1490–2861	1102–11,513
Disease control	days		
evolutive (*N* = 94)	1 (1–3)		*p* value = 0.238
partial response (*N* = 2)	1 (1–1)		
G-CSF prophylactic administration	days		
yes	1 (1–3)		*p* value = 0.598
no	1 (1–3)		

G-CSR = granulocyte-colony-stimulating factors; IQR = interquartile range; CI = confidence interval.
